# Application of a pig ligated intestinal loop model for early *Lawsonia intracellularis *infection

**DOI:** 10.1186/1751-0147-52-17

**Published:** 2010-02-24

**Authors:** Torsten S Boutrup, Kirsten Schauser, Jørgen S Agerholm, Tim K Jensen

**Affiliations:** 1 National Veterinary Institute, Technical University of Denmark, Bülowsvej 27, DK-1790 Copenhagen V, Denmark; 2 Department of Veterinary Disease Biology, Faculty of Life Sciences, University of Copenhagen, Ridebanevej 3, DK-1870 Frederiksberg C, Denmark; 3 Department of Basic Animal and Veterinary Sciences, Faculty of Life Sciences, University of Copenhagen, Grønnegårdsvej 7, DK-1870 Frederiksberg C, Denmark

## Abstract

**Background:**

Porcine proliferative enteropathy in pigs is caused by the obligate, intracellular bacterium *Lawsonia intracellularis*. *In vitro *studies have shown close bacterium-cell interaction followed by cellular uptake of the bacterium within 3 h post inoculation (PI). However, knowledge of the initial *in vivo *interaction between porcine intestinal epithelium and the bacterium is limited. The aims of the present study were to evaluate the usefulness of a ligated small intestinal loop model to study *L. intracellularis *infections and to obtain information on the very early *L. intracellularis*-enterocyte interactions.

**Methods:**

A ligated small intestinal loop model using three different *L. intracellularis *inocula was applied to 10-11-week-old pigs. The inocula were 1) wild type bacteria derived from overnight incubation of *L. intracellularis *bacteria from spontaneous disease, 2) crude vaccine bacteria (Enterisol^® ^Ileitis Vet), and 3) vaccine bacteria propagated in cell culture. The bacteria-enterocyte interaction was visualised using immunohistochemistry on specimens derived 1, 3 and 6 h PI respectively.

**Results:**

Although at a low level, close contact between bacteria and the enterocyte brush border including intracellular uptake of bacteria in mature enterocytes was seen at 3 and 6 h PI for the vaccine and the propagated vaccine inocula. Interaction between the wild-type bacteria and villus enterocytes was scarce and only seen at 6 h PI, where a few bacteria were found in close contact with the brush border.

**Conclusions:**

The ligated intestinal loop model was useful with respect to maintaining an intact intestinal morphology for up to 6 h. Furthermore, the study demonstrated that *L. intracellularis *interacts with villus enterocytes within 3 to 6 h after inoculation into intestinal loops and that the bacterium, as shown for the vaccine bacteria, propagated as well as non-propagated, was able to invade mature enterocytes. Thus, the study demonstrates the early intestinal invasion of *L. intracellularis in vivo*.

## Introduction

The bacterium *Lawsonia intracellularis *is the infectious cause of proliferative enteropathy (PE) in pigs and a range of other animal species [1,2]. The bacterium is Gram negative, rod-shaped and belonging to the delta division of the Proteobacteria. Bacterial growth requires an intracellular environment and *in vitro *isolation and cultivation depends on cell culture [3]. The successful isolation and growth of the bacterium *in vitro *has established the basis for vaccine development [4,5]. Knowledge on the initial host-pathogen interaction *in vivo *is limited. However *in vitro *studies have shown close bacterium-cell interaction followed by cellular uptake of the bacterium within 3 h post inoculation (PI) [6]. Recently experimental infection of pigs has demonstrated enterocyte-bacterium interaction as early as 12 h PI [7].

Intestinal loop models have previously demonstrated their usefulness in studies of *Brachyspira hyodysenteriae *and *Salmonella *Typhimurium [8-11]. McOrist *et al. *[12] used ligated intestinal loops to investigate events between *L. intracellularis *and enterocytes at 1 h PI but found no intracellular uptake of *L. intracellularis *or bacteria-enterocyte interactions. The aims of the present study were to evaluate the usefulness of an intestinal loop model to investigate *L. intracellularis *infections and to obtain information on very early *L. intracellularis*-enterocyte interactions. Compared to the study performed by McOrist *et al. *[12] the exposure time between *L. intracellularis *and the intestinal epithelium in the loops were extended to 1, 3 and 6 h. Moreover three different preparations of *L. intracellularis *inoculums were used at each point.

## Materials and methods

### Experimental animals

Four pigs were purchased from a high health (specific pathogen free (SPF)) herd considered to be free of *L. intracellularis *infection after a medicated eradication program. Twenty blood samples and 10 faecal samples from pigs with body weights (BW) of 30 to 60 kg were sampled twice from the herd and tested by ELISA and PCR methods as described elsewhere [13,14] to ensure herd status regarding *L. intracellularis *infection. All samples tested negative.

The pigs were acclimatised for 2 weeks before entering the study. Clinical signs of disease were not observed during this period. As a precaution, all pigs were medicated with tiamulin at arrival (Tiamutin^® ^vet. 200 mg/ml, Novartis, Copenhagen, Denmark), given at a dosage of 20 mg/kg BW intramuscularly for 4 consecutive days. Faecal samples taken before and after medication were all found negative for *L. intracellularis *by PCR. To avoid adverse effect of the antibiotic treatment on the study, treatment with tiamulin was ceased at least 7 days before inoculation.

The four pigs were housed together and fed a standard diet *ad libitum *(DLG, +10, Aarhus, Denmark) with free access to water and straw. The animals were fasted from the day before experimentation with free access to water with glucose added. The pigs were 10-11-week-old (BW 26 to 31 kg) at the time of surgery. The experimental study was approved by the Danish Animal Experiments Inspectorate under the Ministry of Justice.

### Inoculum

#### Infectious materials derived from spontaneously diseased pigs

Prior to the trial, porcine small intestines having PE were collected from a herd that had previously delivered infectious materials for successful experimental infections [7,15]. The presence of *L. intracellularis *associated with PE in the material was confirmed by immunofluorescense (IF) using an anti-*L. intracellularis *monoclonal antibody (Law1-DK) [16,17]. The intestines were frozen at -80°C in portions of 100 g. The day before inoculation, a portion was thawed in a water bath at 37°C and epithelial cells were isolated by immersing the material into 100 ml of Hank's balanced salts solution (HBSS) without CaCl_2 _and MgCl_2_(Invitrogen, 14180-046, Taastrup, Denmark) diluted 1:10 in Milli Q water, with 5 mM EDTA (Merck, 15498, Albertslund, Denmark) and incubated at 37°C for 80 min with occasional stirring. Detached epithelial cells and *L. intracellularis *bacteria were harvested by centrifugation at 5000 *g *for 30 min. The cells were resuspended in 100 ml Dulbecco's Modified Eagle medium (DMEM) (Invitrogen, 41965) with 5% fetal bovine serum (FBS) (Sigma, F9665, Vallensbaek, Denmark), 1% L-glutamine (Invitrogen, 25030), 2% amphotericin B (Sigma, A2942), gentamycine 50 μg/ml (Sigma, G3632) and vancomycine 100 μg/ml (Sigma, V2002) and incubated overnight at 37°C, in an atmosphere of 8.8% CO_2 _and 8.0% O_2_. Next day the inocula were centrifuged at 5000 *g *for 30 min and resuspended in 50 ml of DMEM with 5% FBS and the epithelial cells were lysed by forcing the suspension through a 3.5 inch 22 Gauge spinal syringe (Becton Dickinson, 405256, Madrid, Spain). *In vitro *cell culture inoculations have shown an initial intracellular replication of similar level using this method compared to crude mucosal scraping (data not shown). Compared to crude mucosal scraping, the described method provides rather homogenous inoculum.

#### Infectious materials derived from commercial L. intracellularis live vaccine

A commercial *L. intracellularis *live vaccine (Enterisol^® ^Ileitis Vet., No. 024390, Batch no 30496-00) was purchased and held at 5°C until use. Immediately before inoculation into intestinal loops 0.8 g of freeze dried vaccine were dissolved in 5 ml of DMEM with 5% FBS. This corresponds to four doses according to manufacturer.

#### Infectious materials derived from commercial L. intracellularis live vaccine propagated in cell culture

Infected cell cultures based on the *L. intracellularis *vaccine were produced by suspending 0.4 g of freeze dried vaccine in DMEM with 5% FBS and 1% L-glutamine and inoculating the suspension into a McCoy cell culture (ATCC number: CRL-1696), T-80 bottles with 15 ml medium seeded with 2 × 10^5 ^cells per ml from the day before. The infected cell cultures were incubated at 37°C, in 8.8% CO_2 _and 8.0% O_2_. Passage of infection was done by scraping of McCoy cells, which were lysed by forcing the suspension through a 3.5 inch 22 Gauge spinal syringe. Cell debris were removed by centrifugation at 150 *g *for 5 min, bacteria were harvested by centrifugation at 5000 *g *for 20 min. The bacterial pellet was re-suspended in 3 ml of medium and re-inoculated onto new cell cultures as described above. At the day of inoculation, two cell culture bottles with massive growth of *L. intracellularis *were used. The cells were scraped from the bottom and lysed as described above. Cells and bacteria were centrifuged at 5000 *g *for 20 min, where after the pellet was re-suspended in 10 ml of medium.

The concentration of *L. intracellularis *in the different inocula was determined by serial 1:10 dilutions in sucrose potassium glutamate (SPG) with 5% FBS. Ten μl of each dilution were added to each well in a six-well glass slide and examined by indirect IF [16]. The number of *L. intracellularis *bacteria was counted at 40× objective magnification in 10 view fields corresponding to 1/25 of a well. The concentrations in the different types of inocula are shown in Table 1. Five ml of each inoculum was injected into the lumen of intestinal loops via an 18 G syringe.

**Table 1:** Overview of types- and concentrations of inocula used in each ligated intestinal loops.

Loop No.	Inoculation time	Inoculation type	Inoculum concentration
1		Wild-type	4-6 × 10^8 ^bacteria/ml
		
2		Live vaccine	3-5 × 10^6 ^bacteria/ml
		
3		Propagated live vaccine	2-8 × 10^7 ^bacteria/ml
		
4		Negative control	Mock inoculum

5		Wild-type	4-6 × 10^8 ^bacteria/ml
		
6		Live vaccine	3- 5 × 10^6 ^bacteria/ml
		
7		Propagated live vaccine	2-8 × 10^7 ^bacteria/ml

8		Wild-type	4-6 × 10^8 ^bacteria/ml
		
9		Live vaccine	3-5 × 10^6 ^bacteria/ml
		
10		Propagated live vaccine	2-8 × 10^7 ^bacteria/ml

Ligation of ten loops (1-10) was done in the ileum and the jejunum, respectively. All three types of inocula applied were exposed for 1, 3 and 6 h, while only at 6 h a negative control was included (loop No. 4). The concentration of *Lawsonia intracellularis *in the inocula is shown in the table; 5 ml of inoculum were used for each loop.

#### Anaesthetic and surgical procedure

Isoflurane inhalation anaesthesia and surgical procedures were done as described by Grøndahl *et al*. [18] and modified by Shauser *et al. *[10]. Isotonic saline was administered intravenously throughout the procedure. Pulse, blood pressure, rectal temperature and blood gas pressure were monitored. A midline abdominal incision was made and ten loops were produced in the upper jejunum and lower jejunum, respectively (Table 1). The first loop in the lower jejunum was made 10 cm oral to the ileocaecal orifice with additional nine loops ligated in oral direction. The first upper jejunal loop was made 1 m oral to the confluent ileal Peyer's patch with additional nine loops ligated in oral direction. Each loop was approximately 5 cm long followed by an inter-loop segment of around 2 cm. Ligation was done by a intestinal circumferential ligature through the mesentery without damaging grossly visible mesenteric vascular arcades thus maintaining full blood supply for both loops and inter-loop segments. The overall anaesthetic period was 7 to 8 h where after pigs were euthanised by an overdose of sodium pentobarbital while still anaesthetised.

Loops were inoculated for 1, 3 and 6 h for each inoculum. Initially four lower jejunal and four upper jejunal loops were made (Table 1). One loop served as negative control and were inoculated with DMEM with 5% FBS, one loop was inoculated with the wild-type bacterial suspension, one with vaccine suspension and one with the suspension of cell culture propagated vaccine. This procedure was repeated after 3 h and again after 5 h, but without control loops (Table 1). Inter-loop segments served as non-inoculated controls at 3 and 5 h.

### Tissue processing

The loops were sampled at euthanasia by cutting the mesentery and immediately cooled on thawing ice. The ends of each loop were cut off, the lumen was rinsed with isotonic saline and the tissues were fixed in 10% neutral buffered formalin for 24 to 48 h. The tissue was cut into transverse sections, exposed to graded series of alcohol succeeded by xylene and embedded in paraffin.

### Immunohistochemistry

The loop specimens, each consisting of two full cross sections, were cut in 5 μm thick sections and mounted on Super Frost*/plus slides (Menzel-Gläser, Braunschweig, Germany). Mounted slides were heated to 60°C, deparaffinised and rehydrated in xylene, graded series of alcohol and finally in water. Endogenous peroxidase activity was inhibited by incubation with 0.6% H_2_O_2 _in tris buffered saline (TBS) (50 mM Tris, 150 mM NaCl, pH 7.6) for 20 min followed by washing in TBS 3 × 5 min. Slides were incubated with 0.05% protease (Sigma, type XXIV, 8038) in TBS for 10 min followed by washing in TBS 3 × 5 min. Slides were incubated 1 h with polyclonal rabbit anti *L. intracellularis *antibody [7] diluted 1:10000 in TBS, washed for 3 × 5 min in TBS and incubated with Envision^+^goat anti-rabbit conjungate (DAKO, K4002, Glostrup, Denmark). After washing for 3 × 5 min in TBS, reaction was developed for 15 min with a solution of 3-amino-9-ethylcarbozole (AEC) (Kementec, 4190, Copenhagen, Denmark) followed by washing in TBS 3 × 5 min, counterstained by Mayer's haematoxylin and mounted with glycergel (DAKO, C563). All procedures were undertaken at a room temperature around 20°C.

### Microscopic evaluation

Slides were evaluated by light microscopy using 40× and 63× objectives. In tissue from mock inoculated loops and inter-loop segments, absence of *L. intracellularis *antigens were evaluated for both intestinal lumen and mucosa.

**Figure 1:**
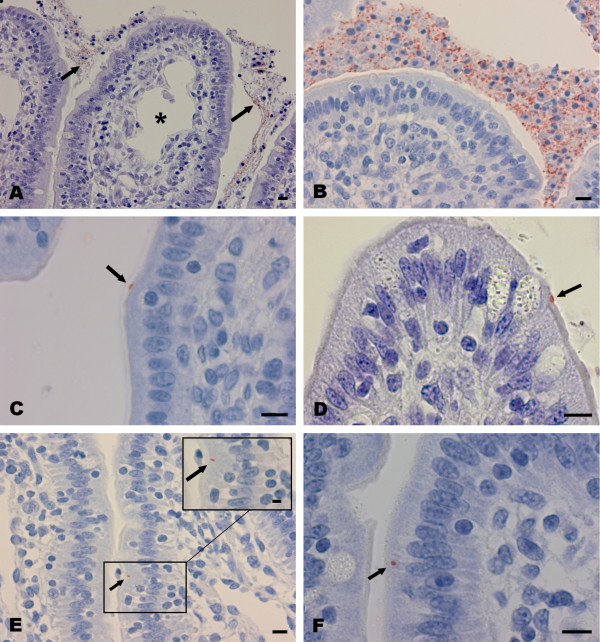
**Visualisation of *Lawsonia intracellularis *in tissue of inoculated intestinal loops**. Immunohistochemistry/haematoxylin stain of *Lawsonia intracellularis *in intestinal tissue; arrows point at immunopositive red stained *L. intracellularis*. A and B: Bacteria overlying ileal epithelium 6 h post inoculation (PI). A) Vaccine derived inoculum. B) Wild-type derived inoculum. In both (A) and (B) close interactions between bacteria and enterocytes is not found. Low level oedema seen as distended central lacteal (A) (asterisk). C and D: Solitary *L. intracellularis *bacteria in intimate contact with the brush border of enterocytes 6 h PI. C) Vaccine derived inoculum in jejunal loop. D) Cell culture propagated vaccine in ileal loop. E and F: Solitary intracellular *L. intracellularis *bacteria in villus enterocytes 6 h PI. E) Vaccine derived inoculum in jejunal loop. F) Cell culture propagated vaccine in ileal loop. Insert in (E) shows a higher magnification of the area with the intracellular bacterium. Bars: 10 μm.

In inoculated loops the presence of intracellular bacteria was evaluated, including a specific search for bacteria in the brush border with no free space in between enterocytes and the bacterium (Figure 1C and 1D). The presence of *L. intracellularis *antigen in the intestinal lumen and mucus overlying villus epithelium and in the crypts was noted but considered as a passive presence due to inoculation.

## Results

Ligation was found to induce grossly visible local mesenteric oedema and decreased intestinal wall tonus. Pallor of the intestinal wall indicating inadequate blood supply did not occur, congestion of mesentery and intestinal vessels remained at a low level and mild stromal oedema was the only histologically circulatory associated lesion (Figure 1A). Together these findings indicate a limited negative impact on the intestinal blood supply due to the procedures applied. In general the rectal temperature was slowly decreased from around 37.5°C to 36.2°C, although one pig had a terminal rectal temperature of 35.8°C. One pig had a mild local chronic adhesive fibrous peritonitis. *L. intracellularis *antigen was not found by IHC in the negative control loops or in the inter-loop segments.

Although only a few bacteria were seen in direct contact with enterocytes or the brush border during the first 6 h PI for all types of inocula, differences were observed as bacteria of the vaccine inoculum and vaccine propagated inoculum seemed to be in direct contact with the mucosa more frequently than the wild type. Bacteria were seen as single distinct organisms within in the brush border of the villus enterocytes 3 h and 6 h PI (Figures 1C and 1D). The number of bacteria in direct contact with the brush border varied but mostly 10-25 organisms per full transverse intestinal section were seen. In addition, single intracellular *L. intracellularis *bacteria (1-5 organisms per intestinal cross section) were found in villus enterocytes 6 h PI (Figures 1E and 1F) indicating a low level infection. By contrast, only 5-10 *L. intracellularis *bacteria of the wild type were seen in close proximity to the brush border for loops inoculated for 6 h but not for loops inoculated for 1 or 3 h. Wild type intracellular bacteria were not observed at all.

Interaction between bacteria and crypt epithelium was not observed irrespectively of type of inoculum. However, IHC demonstrated that the inoculated material had remained in the lumen.

## Discussion

The study demonstrates that mature enterocytes are infected by *L. intracellularis *thus, confirming previous studies examining the bacterium-enterocyte interaction during later stages of infection. In a recent study by Boutrup *et al. *[7]*L. intracellularis *was demonstrated in villus enterocytes 12 h PI in pigs inoculated by stomach tube with a mucosal scraping obtained from pigs naturally affected by PE. Whether *L. intracellularis *is able to propagate in the mature amitotic enterocytes is however not known. Interestingly, invasion was only shown for vaccine derived *L. intracellularis*, cell culture propagated as well as non-propagated. Interaction between bacteria and mucosa was observed at 3 and 6 h PI. Similar to the study by McOrist *et al. *[12] based on a modified intestinal loop model inoculated with a laboratory attenuated strain of *L. intracellularis*, we did not observe interaction between bacteria and enterocytes 1 h PI. It could be postulated that the observed interaction occurred just by chance, i.e. that some bacteria passively adhered to the brush border. However, if that had been the case we would have expected such a phenomenon to occur randomly in all loops. We did not see close interaction at all 1 h PI despite the type of inoculum. Furthermore, differences were observed among inocula as the wild type showed less interaction than the vaccine regarding both the number of interacting bacteria and interaction 3 h PI. This indicates that interaction was not an accidental event.

Direct evidence for specific target cells during the initial exposure of the intestinal epithelium to *L. intracellularis *has not been shown. However, data from experimental studies [19-22] on the location and events of *L. intracellularis *infection from 24 h to 3 wks in hamsters and pigs report the presence of intracellular bacteria and the development of hyperplastic lesions as taking place from infected crypt cells. Also some authors propose the crypt cells to be the target cell population for *L. intracellularis *[23,24]. Bacterial invasion of crypt enterocytes was not observed in this study. However, this may be due to retention of the inoculum above the crypt-villus junction.

The ligated intestinal loop model has previously shown its usefulness in studies of intestinal bacterial infections [8-11]. The validity of the model highly depends on conservation of a normal intestinal function and environment. Our study shows that the model seems useful with respect to maintaining an intact intestinal morphology as the only histomorphological change in the intestinal mucosa seen after ligation of intestinal loops for up to 6 h was a slight stromal oedema. As lethal or sublethal changes, as e.g. hydrophic degeneration or enhanced exfoliation of enterocytes, did not occur, we suggest that the intestinal barrier remained intact and mimicked the epithelium of a non-ligated intestine. However, we cannot exclude the presence of ultrastructural changes of e.g. the cytoskeleton, which might play a role for uptake of bacteria and intracellular replication [25]. However, the model may have several pitfalls. The uneven distribution of the inoculum may indicate an impaired intestinal motility. Also the intestinal microenvironment may have been influenced as a 5 ml inoculum was injected into ligated segments thus arresting normally occurring bacteria and their metabolic products in a confined space. Although not being associated with significant lesions, the ligation may have affected vasculature and nerves causing a change in e.g., pH and oxygen tension in the microenvironment. It cannot be excluded that such physical and/or chemical changes may have affected the properties of *L. intracellularis*. The low level of infection is however surprising, especially because a well established infection is established no later than 12 h PI of infectious material by stomach tube [7] and because the bacteria were in active growth as observed by direct microscopy of cell cultures. The causes remain speculative. The microenvironment may have been unfavourable for both bacteria and enterocytes as discussed previously e.g. the course of an infection with *L. intracellularis *depends on feeding strategies [15,26,27] indicating an importance of intestinal microenvironment on the bacteria. Also the bypassing of the stomach may have influenced the pathogenic potential of the bacteria.

The observed patterns of localisation for the wild-type and vaccine derived *L. intracellularis *differed as the wild-type seemed less infective than the vaccine. This is surprising as the wild-type was supposed to be more virulent. The difference may be due to the procedures used for isolation of the wild-type bacteria. For example, HBBS/EDTA treatment or the addition of antibiotics to the growth medium may have impeded the wild-type. Therefore, this study can not be used for comparison of virulence but only to study the early pathogenesis.

Based on several experiments, it is our experience that induction of clinical disease (diarrhoea, loss of weight and extensive proliferative lesions) following oral inoculation with *L. intracellularis *in pigs older than 6-8 weeks is difficult. This observation is supported by Mapother *et al. *[28], which produced severe gross lesions in pigs weighing around 7 kg but only mild lesions in larger pigs weighing around 55 and 90 kg. The pigs used in the present study were 10-11-week-old at the time of the surgical procedure. Even though others have reported the induction of experimental infection in pigs being 10- week-old [29] or older [30], we believe that an additional study using younger pigs should be performed to evaluate whether this could increase the magnitude of bacteria-enterocyte interaction, and thereby the usefulness of the model.

## Conclusions

The study shows that as early as 3 to 6 h after inoculation into intestinal loops, *L. intracellularis *interacts with villus epithelium resulting in subsequent uptake in mature enterocytes. Furthermore, this study shows the usefulness of a pig ligated intestinal loop model as an alternative to *in vitro *models in investigating early bacteria-host cells interactions in *L. intracellularis *infections. However the limited number of bacteria seen in close association with or intracellular in enterocytes limits the models usefulness with regard to investigating factors enhancing or blocking cellular uptake.

## Competing interests

The authors declare that they have no competing interests.

## Authors' contributions

TSB designed the study, prepared the inoculum, performed the surgical procedures, sampled materials, did the initial histopathological and immunohistochemical evaluations, participated in interpretation of results and drafted the manuscript. KS participated in designing the study and participated in the surgical procedures and drafting of the manuscript. JSA and TKJ participated in designing the study, interpretation of results and drafting of the manuscript. All authors read and approved the final manuscript.
